# Strengthening Biorisk Management Capacities in Burkina Faso: Contribution of the Global Health Security Agenda

**DOI:** 10.1089/hs.2019.0069

**Published:** 2022-12-08

**Authors:** Emilie Dama, Arnaud Orelle, Abdoulaye Nikiema, Patrick D. Mandingar, Alphonse Naby, Gérard B. Bationo, Mah-Séré Kéita, Antoine Pierson, Charles Sawadogo, Rebecca Greco Koné

**Affiliations:** Emilie Dama, PhD, MSc, is Senior Laboratory Advisor, the Burkina Faso Country Office, US Centers for Disease Control and Prevention, Ouagadougou, Burkina Faso.; Arnaud Orelle, MSc, was Biosafety and EQA Program Coordinator, Integrated Quality Laboratory Services, Villeurbanne, France.; Abdoulaye Nikiema, PharmD, MSc, is GHSA Program Manager, African Society of Laboratory Medicine, Ouagadougou, Burkina Faso.; Patrick D. Mandingar, PharmD, MSc, is Head, Monitoring, Evaluation and Quality Management Department, the Directorate of Biomedical Laboratories, General Directorate of Access to Health Products, Ministry of Health, Ouagadougou, Burkina Faso.; Alphonse Naby is Biomedical Technician, the Directorate of Biomedical Laboratories, General Directorate of Access to Health Products, Ministry of Health, Ouagadougou, Burkina Faso.; Gérard B. Bationo, PharmD, DES, is Head, department in charge of medical laboratories monitoring, the Directorate of Biomedical Laboratories, General Directorate of Access to Health Products, Ministry of Health, Ouagadougou, Burkina Faso.; Kéita Mah-Séré, MPH, is Director of Programs, African Society of Laboratory Medicine, Bamako, Mali.; Antoine Pierson, PharmD, is Chief Scientific Officer, Integrated Quality Laboratory Services, Villeurbanne, France.; Charles Sawadogo, PharmD, CES, is Director of Biomedical Laboratories, the Directorate of Biomedical Laboratories, General Directorate of Access to Health Products, Ministry of Health, Ouagadougou, Burkina Faso.; Rebecca Greco Koné, MSc, is Director, the Burkina Faso Country Office, US Centers for Disease Control and Prevention, Ouagadougou, Burkina Faso.

**Keywords:** Biorisk management, Global Health Security Agenda, Laboratory assessment, Laboratory safety, Burkina Faso

## Abstract

The Global Health Security Agenda and the International Health Regulations (2005) recommend that countries strengthen the capacity of their national laboratory systems to comply with the International Health Regulations. To efficiently and effectively direct these efforts, the US Centers for Disease Control and Prevention—in collaboration with the Ministry of Health Directorate of Laboratories, the African Society for Laboratory Medicine, and Integrated Quality Laboratory Service—assessed Burkina Faso's national laboratory system using the World Health Organization *Laboratory Assessment Tool*. Based on gaps observed in biorisk management, the Biosafety and Biosecurity Laboratory Assessment Tool (BSS LAT) was developed to assess 10 public laboratories handling dangerous pathogens. This tool uses a specific scoring matrix with quantitative output. Composite assessment scores for the 9 primary modules (capacity areas) were reported, with the highest scores reported in cleaning, disinfection, sterilization, waste management (42%), and good laboratory practices (40%), and the lowest scores in biosecurity/biosafety (33%), documents/regulations (18%), emergency management (16%), and risk management (5%). To address challenges identified in the assessments, a set of activities was planned with a focus on biorisk management. Results from an evaluation conducted 1 year later, using the BSS LAT, showed an increase in the average score of all indicators from 25% to 45% and an increase in the biorisk management module score from 5% to 35%. This evaluation process was a decisive step toward strengthening the capacity of the laboratory system in Burkina Faso. Global Health Security Agenda investments and activities have made a lasting impact on improving biosafety and biosecurity in public health laboratories. To ensure sustainability, a strong laboratory quality management program based on a mentorship system is greatly needed.

## Introduction

The World Health Organization (WHO) International Health Regulations (IHR) 2005^1^ are a legally binding international agreement designed to prevent the spread of disease. The purpose and scope of the IHR are “to prevent, protect against, control, and provide a public health response to the international spread of disease in ways that are commensurate with and restricted to public health risks, and which avoid unnecessary interference with international traffic and trade.”^1^ To that end, indicator 13 in the IHR checklist—“Laboratory biosafety and laboratory biosecurity (biorisk management) practices are in place”^2^—requires that countries establish laboratory biosafety and biosecurity practices to prevent the exposure of laboratory staff, the public, and the environment to potentially dangerous pathogens and other biological hazards. Biosafety and biosecurity are complementary and are understood as follows:

*[B]iosafety refers to principles, technologies, practices and measures implemented to prevent the accidental release of, or unintentional exposure to pathogenic agents; biosecurity refers to the protection, control and accountability measures implemented to prevent the loss, theft, misuse, diversion or intentional release of pathogenic agents and related resources, as well as unauthorized access to, retention, or transfer of such material.^3^*

The rapid spread of Ebola virus disease across West Africa in 2014-2016 and SARS-CoV-2 globally provided tangible demonstrations of the low capacity in limited-resource countries to control infectious disease outbreaks and associated biosafety and biosecurity risks.^4-7^ In the current context of globalization, strengthening the capacities of resource-limited settings is even more urgent to better control epidemics and safeguard all populations.

To support countries in their efforts to comply with the IHR, the Global Health Security Agenda (GHSA) Commitment Development meeting held in Helsinki, Finland, in May 2014 resulted in the identification of 11 GHSA Action Packages^7^ (or technical areas) organized around the need to prevent, detect, and respond to public health threats. These Action Packages include biosafety and biosecurity (prevent) and the national laboratory system (detect). Known as essentials pillars of international health security,^8^ the purpose of the biosafety and biosecurity package under GHSA is to reduce the capability of dangerous pathogens to spread rapidly within and across borders.

In recent years, considerable resources have been invested in improving laboratory systems in limited-resource countries, including infrastructure, diagnostics, and equipment.^9^ More coordination is needed, however, to implement national laboratory plans, improve the quality of laboratory services, and ensure appropriate biosafety and biosecurity conditions and practices for handling dangerous agents to effectively move toward a world that is safer and more secure from infectious disease threats.^10-12^ With the COVID-19 pandemic, biosafety and biosecurity measures focused on One Health aspects of the disease outbreak and SARS-CoV-2 spread were considered key points to restraining this pathogen.^13^

Burkina Faso is 1 of 17 high-priority countries supported by the US Centers for Disease Control and Prevention (CDC) under the GHSA.^14^ Laboratory system strengthening is a high priority for the Burkina Faso government and a key focus of CDC's support in the country. It is worth noting that before initiating the GHSA biorisk management program, no recent data existed on the performance of laboratories that had used effective evaluation tools to identify gaps to be addressed. To achieve its goal, the CDC's global health security support in Burkina Faso has relied on existing country assets, such as the infrastructure and human resources of the Ministry of Health's Technical Directorate of Laboratory, the National Laboratory Strategy, and key guidance documents,^15-18^ including standard operating procedures and policies. These documents cover quality control in biomedical laboratories, the nomenclature of medical biology, good laboratory practices, infrastructure and equipment, and maintenance. Despite the existence of these resources, little had been done related to implementing a biorisk management program in Burkina Faso before the government engaged with the GHSA. It is also important to note that the risk of infectious disease outbreaks and bioterrorist attacks has grown in recent years as terrorism threats have increased.^19^ The responsibilities of the National Biosecurity Agency (ANB) of Burkina Faso are currently limited to biotechnologies and genetically modified organisms, and, therefore, the initiative to implement a broader, institutionalized biorisk management program through the GHSA was welcomed. This program has enabled the government of Burkina Faso to strengthen its capacity and minimize safety and security risks associated with the handling, storage, and disposal of biological agents and toxins in the country's main laboratories, in accordance with national and international requirements.^9,11,20,21^

To this end, the CDC—in collaboration with the Ministry of Health Directorate of Laboratories, the African Society for Laboratory Medicine, and consulting agency Integrated Quality Laboratory Service (IQLS)—conducted an assessment of the national laboratory system, using a tailored version of the WHO *Laboratory Assessment Tool* (WHO LAT).^20^ Based on gaps identified during this assessment, a roadmap was developed to strengthen Burkina Faso's biosafety and biosecurity program within the laboratory system.

In this article, we report on the process, achievements, and improvement of biosafety and biosecurity capacity in 10 laboratories handling dangerous pathogens in Burkina Faso. We then discuss the impact of capacity strengthening efforts and lessons learned related to compliance with the IHR.

## Methods

The approach used by the GHSA to strengthen biosafety and biosecurity capacity in Burkina Faso can be divided into 4 main steps: (1) assessment, (2) roadmap development, (3) implementation of the roadmap activities, and (4) evaluation. To do this, the African Society for Laboratory Medicine hired IQLS, with financial and technical support from CDC, to conduct a laboratory systems assessment in Burkina Faso in line with GHSA requirements and to develop roadmaps to define the way forward for meeting the GHSA requirements for the national laboratory system, mainly focused on biosafety and biosecurity. The assessment step evaluated 2 tools: a tailored version of the WHO LAT and the newly developed Biosafety and Biosecurity Laboratory Assessment Tool (BSS LAT).

### WHO LAT Assessment

The initial 4-day assessment of the national laboratory system took place in July 2016 using a tailored version of the WHO LAT.^20^ This tailored tool included 8 modules (capacity areas): (1) governance, (2) organization and structure, (3) quality management system, (4) human resources, (5) equipment and supplies, (6) information management, (7) biorisk management, and (8) communication within the network (Supplemental Appendix 1, www.liebertpub.com/doi/suppl/10.1089/hs.2019.0069). Each module contains several indicators. The biorisk management indicator focused on the organization of biosafety and biosecurity at a national level, such as the availability of guidelines, policies, and regulations, with only a few questions dedicated to laboratories. The assessment process included meeting with an in-country assessment committee, composed of representatives from the directorate of biomedical laboratory within the Ministry of Health and several tiers of the laboratory network. During the assessment, the committee scrutinized the tool, discussed each question extensively, and reached consensus on the scores.

### BSS LAT Development and Assessment

Based on results from the initial assessment, which showed poor performance of the biorisk management module, the team developed the standardized BSS LAT to specifically assess biosafety and biosecurity practices in individual laboratory facilities. This new tool included 9 primary modules: (1) premises and workflow; (2) staff management and training; (3) good laboratory practices, including personal protective equipment and biosafety cabinets (BSC); (4) cleaning, disinfecting, sterilization, and waste management; (5) emergency management; (6) risk management; (7) documentation and regulations; (8) biosecurity; and (9) other risk management (eg, electrical, chemical) (Supplemental Appendix 2, www.liebertpub.com/doi/suppl/10.1089/hs.2019.0069). Two additional optional modules, one focused on biosafety level 3 laboratories and one on veterinary-specific diseases, were included in the tool, but are not discussed here. Like the WHO tool, each module contained several indicators.^22^ The BSS LAT was then implemented in selected laboratories to determine their baseline capacities in biosafety and biosecurity.

In April 2017, 2 teams conducted a 5-day assessment using the BSS LAT in 10 national public health and regional biosafety level 2 laboratories, which handle and store potentially dangerous pathogens. The national reference laboratories for tuberculosis, meningitis, HIV, influenza, and viral hemorrhagic fevers, including Ebola virus disease, were among the 10 selected laboratories. The assessment process included an introductory meeting with laboratory management, a thorough visit and inspection of laboratory facilities (following the workflow for processing samples), an interview with key staff (to complete a questionnaire), and a meeting to debrief laboratory management. Responses to questions were verified (via additional inspection, documents, and records), and photos were taken during the assessment to include in the report.

Each team, composed of an expert from IQLS and a national assessor, conducted assessments of 5 laboratories. The national assessors were from the Directorate of Laboratory and were given a didactic presentation of the tool and instructions on how to conduct the assessment. Their training process included observing assessments of the first 4 laboratories by experts from IQLS, after which they conducted an assessment under the supervision of experts for the final laboratory.

### Structure of Both Tools

The 2 assessment tools discussed here are structured similarly using modules, with each modules containing a set of indicators. For each indicator, closed questions with possible answers of yes, no, partial (only for WHO LAT), and nonapplicable are included. Based on the responses to these questions, the tool generates a score: answering “yes” gives 100% to the question, answering “partial” gives 50% to the question, answering “no” gives 0% to the question, and answering “nonapplicable” excludes the question from the calculation. Each indicator score is calculated based on the responses to all questions within that indicator. The mean score for each module is the average of all indicator scores in the module. Finally, the general indicator score is the average score of all modules combined, which provides a summary of overall capacity. For the BSS LAT, calculations used the mean score of the 10 assessed laboratories.

### Key Areas for the Roadmap

**Figure 1.**
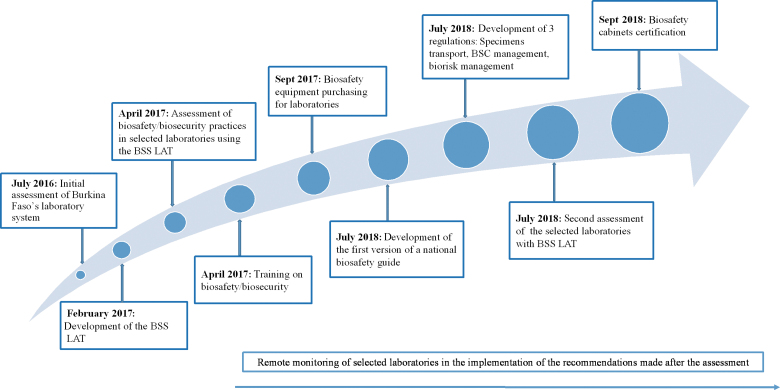
Timeframe of the implementation of activities. Abbreviations: BSS LAT, Biosafety and Biosecurity Laboratory Assessment Tool; BSC, biosafety cabinet.

Based on gaps identified in biosafety and biosecurity during the assessment of the national laboratory system (using the WHO LAT) and of individual laboratory facilities (using the BSS LAT), a set of activities was prioritized for action as part of a 1-year roadmap. This roadmap includes the development of training materials and provision of a training of trainers program on biosafety and biosecurity practices, development of biosafety and biosecurity guidelines and regulations, certification of BSCs in selected laboratories, and procurement of critical biosafety equipment (eg, BSCs, incinerators, uninterruptible power sources) and supplies for the laboratories (Figure 1). A second assessment using the BSS LAT was administered 1 year later by the national assessors from the laboratory directorate to measure the impact of the implemented activities.

**Table 1.** National Laboratory System Assessment Summary Using the World Health Organization Laboratory Assessment Tool

Indicators	Scores %
General indicator score (average of all indicators)	52^a^
Governance	47^b^
System coordination body	72
Policy and strategic plan	53
Services funding	17
Organization and structure	58^a^
Structure	75
Reference laboratories organization	100
Disease-specific reference laboratories	55
Laboratory network(s)	78
Specimen referral system	75
Laboratory regulation mechanism	38
Laboratory/IHR compliance	40
Collaboration with animal, food, and water laboratory system	33
Antibiotic resistance surveillance	30
Quality management system	58^a^
Quality policy and plan	88
Standards for laboratory	70
Disease-specific SOP availability	25
SOP for clinical laboratories	25
Preanalytical documentation	75
External quality assessment	77
Internal quality control	80
Accreditation	33
Supervision	50
Human resources	63^a^
Human resources inventory and availability	43
Laboratory workers' preservice training and qualification	75
Laboratory workers' knowledge/competence, management/retention	77
Human resources motivation and support	58
Equipment and supplies	18^b^
Equipment management	7
Equipment financial resources	17
Supplies management	31
Information management	75^a^
Information management at laboratory level	25
Information notification	100
Information notification tools	75
Feedback/communication	100
Biorisk management	38^b^
Biosafety and biosafety documentation at laboratory level	33
Policies and regulations	57
Good practices in biorisk management at laboratory level	33
Staff occupational work services	30
Communication within the network	56^a^
Communication strategy	0
Support, helpdesk, and advice	58
Laboratory network bulletin	67%
Laboratory network website	33%
Communication possibilities	77%
Professional associations	100%

Note: The capacity score (0%–100%) for each module of the tool is indicated and coded: ^a^indicates acceptable improvement (50%-80%); ^b^indicates a need for major improvement (<50%).

Abbreviations: IHR, International Health Regulations (2005); SOP, standard operating procedure.

## Results

### Laboratory System Assessment Outcomes

The Table provides an overview of the capacity score results, by indicator, of the Burkina Faso national laboratory system, using the 8-module tailored WHO LAT. The general indicator score (average score of all modules) for the national laboratory system was 52%. The modules with the highest scores were information management (75%) and human resources (63%). The lowest scores were in biorisk management (38%) and equipment and supplies (18%). The main challenges related to biosafety and biosecurity at the national level were a lack of guidelines and regulations. At the laboratory level, insufficiencies were also noted in documentation, good practices in biorisk management (eg, equipment, practices, trainings, documents, standard operating procedures), and staff occupational work services.

### BSS LAT Implementation and Training

**Figure 2.**
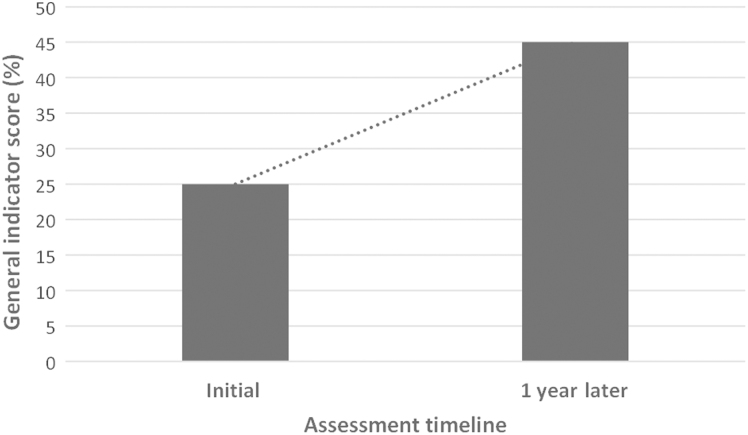
The evolution of the general indicator score between the initial assessment (conducted in 2017) and 1 year later (2018) using the Biosafety and Biosecurity Laboratory Assessment Tool. This score represents the average score of the mean score of the 9 modules.

**Figure 3.**
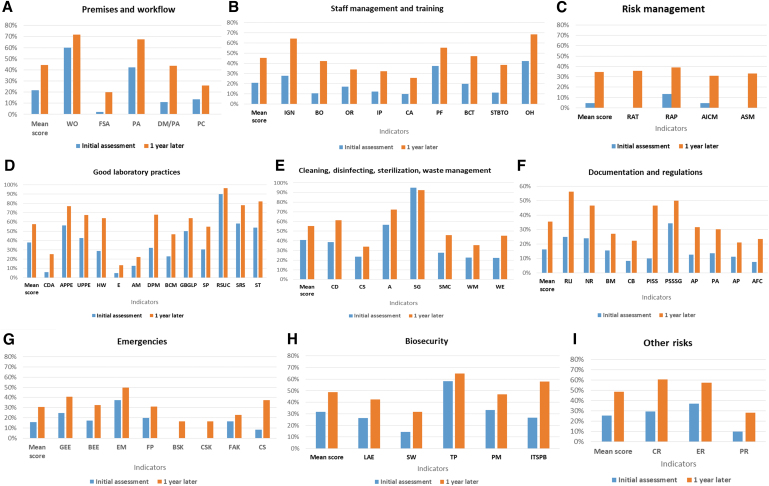
Summary of the results from the initial assessment (conducted in 2017) of biosafety and biosecurity compared with the results 1 year later (conducted in 2018) using the Biosafety and Biosecurity Laboratory Assessment Tool. Indicator abbreviations by category: (A) DM/PA, data management/preanalytical; FSA, facilities and safety assessment; PA, preanalytical; PC, premises control; WO, workflow organization. (B) BCT, biosafety continuous training; BO, biosafety officer; CA, competency assessment; IGK, introduction/general knowledge; IP, induction program; OH, occupational health; OR, organization and responsibilities; PF, personnel file; STBTO, safety training/biosafety before taking office. (C) AICM, analysis and implementation of corrective measures; ASM, audits, surveillance, monitoring: RAP, risk assessment performance; RAT, risk assessment training. (D) AM, aerosol management; APPE, availability of personal protective equipment; BCM, biosafety cabinet management; CDA, clean and dirty areas; DPM, dangerous pathogen management; E, equipment; GBGLP, general behavior/good laboratory practices; HW, handwashing; RSUC, reuse of single-use consumables; SP, sample preparation; SRS, specimen referral system; specimen transportation (ST). UPPE, use of personal protective equipment. (E) A, antiseptics; CD, cleaning and disinfecting; CS, cleaning staff; SG, sterilization in general; SMC, sterilization management and control; WE, waste elimination; WM, waste management. (F) AFC, availability of forms/checklist; AP-E, availability of procedures (emergency); AP-H, availability of procedures (hygiene, cleaning, disinfection); BM, biosafety manual; NR, national regulations; PA, procedure availability (good laboratory practices); PISS, product insert and safety sheets; PSSSG, presampling and sampling SOP/guideline; RLI, relationships of the laboratory with International Health Regulations. (G) BEE, biosafety emergency equipment; BSK, biological spill kit; CS, chemical safety; CSK, chemical spill kit; EM, emergency management; FAK, first aid kit; FP, fire prevention; GEE, general emergency equipment. (H) ITSPB, IT security protection and back up; LAE, laboratory access and evacuation; PM, pathogen management; SW, signage and warnings; TP, theft protection. (I) CR, chemical risks; ER, electrical risks; PR, physical risks.

The BSS LAT was conducted onsite to assess the biosafety level in the 10 main laboratories handling dangerous pathogens at national and regional levels. Following this assessment, a 5-day training on biosafety and biosecurity was provided to 15 staff members from the 10 laboratories. The trainees were selected based on their roles as biosafety and biosecurity focal points. An evaluation showed a 31.7% general increase in knowledge and skills between the pretest and posttest. Scores for the pretest ranged from 33.3% to 88.9% and from 79.6% to 100.0% for the posttest. At the end of the training, each participant developed an action plan to be implemented in their individual laboratories. To strengthen and sustain country capacity, all training materials were shared with the participants, and 2 people from the Directorate of Laboratory were trained on the use of the BSS LAT. This enabled the Ministry of Health to gain the skills and competence to conduct an evaluation in the selected laboratories 1 year after the implementation of the roadmap activities and to compare the results with the initial findings to highlight the impact. BSS LAT results showed an increase in the general indicator score from 25% to 45% between the first and second evaluation (Figure 2). For individual modules, the evaluation showed the highest increase of 30% in biorisk management (with a range of 5% to 35% increase) and the smallest increase of 13% in cleaning, disinfection, sterilization, and waste management. It is worth noting that only 2 modules reached a mean score of at least 50% (the acceptable level) after the second evaluation, indicating that improvement is still needed across most, if not all, of the module areas (Figure 3).

Before engaging with the GHSA to strengthen laboratory biorisk management capacity, no BSC had ever been certified in Burkina Faso. With support from CDC, the African Society for Laboratory Medicine contracted with the Institute of Human Virology, Nigeria, the only National Sanitation Foundation (NSF) accredited BSC certifier in West Africa to travel to Burkina Faso to certify BSCs. Twelve BSCs were selected and have been certified in the 10 selected laboratories according to European EN12469-2000 or US NSF 49 standards for microbiological safety cabinets. Recommendations related to management of BSC include: (1) training operators on use, care, and maintenance of BSC; (2) training national certifiers who will mentor and monitor the operators; and (3) regular annual certification for the BSCs.

One of the weaknesses identified in the laboratory system assessment was an absence of guidelines and regulatory documents to provide a more comprehensive and integrative understanding of biosafety and biosecurity as required by the IHR.^1^ Through a participatory planning approach, IQLS, African Society for Laboratory Medicine, CDC, and Burkina Faso Ministry of Health biosafety experts worked closely to develop and validate the first national guide for security and safety in biomedical laboratories. This guide was intended to be comprehensive, describing the roles and responsibilities of different actors in the whole biosafety system, good practices in biosafety and containment, and procedures to be followed. The guide also includes an overview of facilities and biosafety equipment. Copies of the guide were distributed to all public healthcare facilities at all levels within the healthcare system, as well as national, regional, district, and private laboratories.

In addition, 3 regulations were drafted to support ANB guidance. The first is focused on specimen transport and addresses both the import and export of biological materials. The second is related to risk assessment and presents the components and procedures for risk evaluation in a laboratory. The third is on BSC management and provides technical specifications, conditions of BSC purchasing, installation, and maintenance. These regulations are under review by the ANB, in collaboration with the Directorate of Laboratory, with the purpose of harmonizing them with existing regulations under the agency. Once validated, they are to be endorsed by the ANB, which is the legal guarantor of biosecurity in the country.

## Discussion

Given increased concerns related to the frequency of biological incidents and epidemics in West Africa, biorisk management is a concern for Burkina Faso and all West African countries.^12^ The approach used under the GHSA program in Burkina Faso is to build on the capacities of national laboratory systems assets, identify areas for improvement, and support complementary activities. This enabled the strengthening of the biosafety and biosecurity capacities of Burkina Faso's laboratory system, with an initial focus on laboratories handling high-consequence pathogens.

Similar approaches using a scoring tool or checklist to assess laboratory systems or reference laboratories have been successfully used in sub-Saharan African countries,^23^ including Ghana,^24^ as well as Indonesia,^25^ Malaysia,^26^ and Thailand.^27^ In Burkina Faso, strengthening human resource capacity at the Directorate of Laboratory—focusing on use of the biosafety and biosecurity assessment tool—enabled the country to accurately monitor the implementation of recommendations and the roadmap, which were developed at the end of the training. An important finding was an increase in the general indicator score from 25% to 45% just 1 year after the start of the program. Interestingly, the greatest increase observed was in biorisk management (30%). Noteworthy improvement in this module included risk assessment training (0% to 36%), risk assessment performance (13% to 39%), analysis and implementation of corrective measures (5% to 31%), and audits, surveillance, monitoring (0% to 33%). Because a good risk assessment is critical for a laboratory management system to effectively prevent the occurrence of events, particular emphasis was placed on conducting risk assessments during the biosafety training working sessions. Some laboratories were able to effectively conduct a risk assessment, but this is still an area for continued focus and improvement.^21,28^ Modules such as premises and workflow (22% to 44%), staff management and training (21% to 45%), and good laboratory practices (38% to 58%) showed a moderate increase in scores. A significant observation made during the second assessment was the appointment of biosafety officers in 5 laboratories and an overall improvement in general knowledge on biosafety, infection prevention and control practices, and risk groups. The training and action plans developed by each laboratory may have contributed to this achievement and the improvement was observed in other indicators. As a general note, scores in the remaining 5 modules increased minimally (less than 20%). However, indicators such as relationships of the laboratory with the IHR (25% to 56%) and the development of procedures and safety sheets (10% to 47%) did show improvements.

Before the 2014-2016 Ebola outbreak in West Africa, the capacity to ensure biosafety and biosecurity in reference laboratories of Burkina Faso, as in other West African countries, had never been seriously challenged.^29^ This was due in large part to the lack of initiatives to prevent and reduce potential risks related to the handling and storage of dangerous pathogens. One potential explanation is that in sub-Saharan African countries, resources are more often directed to equipment, whereas few resources are dedicated to biosafety programs.^12^

Certification for BSCs, which are considered the primary containment barriers for manipulation of infectious materials, had never been done in Burkina Faso. Because there are few NSF-accredited BSC certifiers in West Africa, the BSC certification process is difficult and expensive. However, the importance of ensuring strong biosafety and biosecurity programs cannot be understated because pathogens know no borders. Consequently, appropriate actions should be taken to ensure that recommended guidelines are available for proper containment according to the class of pathogen handled. While the current level of biorisk management in Burkina Faso may still be considered “low” under IHR requirements, substantial progress has been made toward preventing or mitigating the impact of naturally occurring outbreaks and accidental or intentional release of dangerous pathogens as a result of efforts under the GHSA program.

A recommendation from the WHO Joint External Evaluation conducted in Burkina Faso in December 2017 was the establishment and strengthening of national legislation and regulations for biological safety and security.^30^ The first edition of the national guide for security in biomedical laboratories and the draft of the 3 regulations are intended to improve practices at all levels of the laboratory system and better control biorisk management. Now, particular emphasis needs to be placed on mobilizing domestic and external financial resources to better sustain these efforts, including efforts toward institutionalizing the relevant policies through approved legislation or regulation.^31^

Although most major laboratories in the country have shown substantial improvements in biosafety and biosecurity performance, improvements in 7 of 9 modules (Figure 3) were lower than the threshold of 50%, which is considered unacceptable. Only good laboratory practices (58%) and cleaning, disinfecting, sterilization, and waste management (55%) scored higher than the 50% threshold. We are aware that our approach may have 2 limitations. The first is the absence of strong remote monitoring to ensure the implementation of recommendations in each laboratory, and the second is that initial GHSA support was focused mainly on reference laboratories, but regional and peripheral laboratories also need reinforced capacity in biosafety and biosecurity.

In summary, efforts under the GHSA to improve Burkina Faso's national laboratory system, with a focus on biosafety and biosecurity, contributed to the identification of strengths and weaknesses. As a result, a roadmap was developed and successfully implemented to address high-priority areas in a comprehensive and practical manner. To our knowledge, this is the first comprehensive biosafety and biosecurity enhancement at the country level targeting several axes of biosafety strengthening within a short time period of 1 year.

## Conclusion

For other countries interested in strengthening biorisk management, the use of an accurate and well-designed assessment tool that not only addresses biorisk management at the national level but also targets challenges at the facility level is vital. If such an assessment tool is mastered at a high level, it could be applied routinely to (1) monitor the biosafety and biosecurity level of laboratories across the country, (2) design interventions, and (3) measure their impact. In addition, guidelines, regulations, and policies should be available at the national level to support practices at the facility level. A strong follow-up system, ideally through a mentorship program and regular supervision, is also necessary to ensure that recommendations from the assessment are implemented. Finally, strong and sustainable biorisk management capacity requires sufficient funding and a national commitment to mobilize resources to consolidate achievements.

## Supplementary Material

Supplemental data

Supplemental data
